# Clinical and Pathological Outcomes of Experimental *Plasmodium fragile* Infection in a Rhesus Macaque

**DOI:** 10.4269/ajtmh.25-0222

**Published:** 2025-10-07

**Authors:** James E. Prusak, Sydney M. Nemphos, Hannah C. Green, Sallie L. Fell, Cecily C. Midkiff, Avelina Rodgers, Jillian Perret, Brooke Grasperge, Krystal Vail, Nana Minkah, Brandon Wilder, Berlin Londono-Renteria, Robert V. Blair, Jennifer A. Manuzak

**Affiliations:** ^1^ Tulane National Primate Research Center, Covington, Louisiana;; ^2^ Tulane University School of Medicine, New Orleans, Louisiana;; ^3^ Center for Global Infectious Disease Research, Seattle Children’s Research Institute, Seattle, Washington;; ^4^ Department of Pediatrics, School of Medicine, University of Washington, Seattle, Washington;; ^5^ Oregon Health and Science University, Portland, Oregon;; ^6^ Tulane University Celia Scott Weatherhead School of Public Health and Tropical Medicine, New Orleans, Louisiana

## Abstract

The anatomical and immunological similarities between humans and nonhuman primates (NHPs) make NHPs a viable model for examining *Plasmodium* spp. infection outcomes. *Plasmodium fragile *was previously used to model human severe malaria in NHPs using rhesus macaques (RMs), but a thorough investigation of the clinical and pathological effects of RM *P. fragile *infection has not yet been conducted. In this study, we observed that experimental infection of a male RM with *P. fragile-*infected erythrocytes resulted in clinical signs of malaria, including anemia; changes in red and white blood cell distribution; changes in red blood cell morphology; and gross and histopathological alterations to vital organs, such as the liver, lungs, heart, and brain. *Plasmodium fragile-*infected red blood cells were also observed in the vasculature of major organs, including the spleen, liver, lung, and brain. These results suggest that experimental *P. fragile* infection of RMs is a translatable model of human malaria.

## INTRODUCTION

In 2023, there were approximately 263 million cases of malaria caused by *Plasmodium *spp*. *infection; among these cases, there were more than 597,000 malaria-related deaths.^1^ Clinically, malaria ranges from uncomplicated to severe, and if untreated, it can result in organ failure, coma, and death.^2^ Several species of primate *Plasmodium*, including *Plasmodium coatneyi*,* Plasmodium cynomolgi*, and *Plasmodium knowlesi*, have been used to investigate the clinical and immunological outcomes of *Plasmodium* infection in nonhuman primates (NHPs), although no NHP model perfectly recapitulates human malaria.^3^^,^^4^ Additionally, direct infection of rhesus macaques (RMs; *Macaca mulatta*) and *Aotus* spp. with *Plasmodium falciparum* and *Saimiri* spp. with *Plasmodium vivax* has been used to study vaccine candidates and novel therapeutics.^3^^,^^4^ *Plasmodium fragile* infection of RMs has been used to model human *P. falciparum *infection because of its ability to undergo antigenic variation, sequester in tissue, and cause nonrelapsing disease and cerebral malaria.^5^^–^^7^ However, detailed reports regarding *P. fragile *pathobiology and clinical outcomes, such as alterations of hematological parameters, in RMs are lacking. Here, we describe the effects of experimental *P. fragile* in a male RM, including longitudinal hematological changes and gross and histopathological findings at necropsy.

## MATERIALS AND METHODS

### Study animal and approval.

One adult (14-year-old), male, *Plasmodium*-naïve, Indian-origin RM was housed and cared for at the Tulane National Primate Research Center (TNPRC) under an Institutional Animal Care and Use Committee (Office of Laboratory Animal Welfare Assurance no. A4499-01)-approved protocol (P0477-3564) in an accredited facility (Association for Assessment and Accreditation of Laboratory Animal Care International no. 000594) compliant with U.S. Department of Agriculture regulations, including the Animal Welfare Act (9 CFR), the Animal Care Policy Manual, the National Research Council in the Guide for the Care and Use of Laboratory Animals, and the Weatherall Report. The criteria established for the inclusion of this RM in the study was no prior exposure to *Plasmodium *spp. This individual RM was considered the experimental unit within the study. Before study assignment, this animal was a part of the TNPRC specific pathogen-free breeding colony and had not undergone any previous procedures. Breeding colony animals are housed in outdoor enclosures in social groups and are free of the following pathogens: simian immunodeficiency virus, simian T-lymphotrophic virus 1, type D simian retrovirus, and *Macacine herpesvirus 1* (MHV1 or B virus). The colony is also monitored for tuberculosis, measles, and additional infectious agents based on risk assessment. After study assignment, the RM was singly housed indoors under climate-controlled conditions with a 12-hour day/12-hour night cycle. Water was available ad libitum, and the RM was fed commercial monkey chow (Purina LabDiet; PMI Nutrition International, Richmond, IN). Anesthesia was performed before all blood draws and inoculation in accordance with TNPRC policy and the Weatherall Report using ketamine hydrochloride (Ketaset, Zoetis, Florham Park, NJ; 6 mg/kg) and buprenorphine (0.01 mg/kg) intramuscularly. Euthanasia was performed at the study end point with intravenous sodium pentobarbital (156 mg/kg) per TNPRC policy. All data points for each outcome described from this RM were included in the analysis.

### *Plasmodium fragile* inoculation and monitoring.

Inoculation with 20 × 10^6^ cryopreserved *P. fragile*-infected red blood cells (iRBCs; Sri Lanka strain; Malaria Research and Reference Reagent Resource Center [now part of BEI Resources, Manassas, VA], MRA-352, lot 226608) was conducted intravenously through the saphenous vein and monitored with Giemsa staining of thin blood smears and quantitative polymerase chain reaction (qPCR) as previously described.^8^^,^^9^ The qPCR reaction volume total was 20 *µ*L using 5 *µ*L of template DNA (Quick DNA/RNA Miniprep Kit; Zymo Research, Irvine, CA), 13 *µ*L of PrimeTime Gene Expression Master Mix (Integrated DNA Technologies [IDT], Coralville, IA) containing concentrations of 300 nM forward and reverse primers and 250 nM probe, and 2 *µ*L of polymerase chain reaction-grade water (IDT). A standard curve of serially diluted *P. fragile *18S DNA gBlocks (IDT) spiked with *P. fragile-*naïve RM genomic DNA along with no-template and no-amplification controls was used in each reaction set. Quantitative polymerase chain reaction results are reported as parasite copies per microliter of DNA.

### Sample collection, processing, and *P. fragile* iRBC cryopreservation.

Peripheral blood was collected in ethylenediaminetetraacetic acid (EDTA) and serum gel vacutainer tubes (Starstedt, Newton, NC) through the femoral vein every 7–8 days and at the clinical end point. Complete blood counts and serum chemistries were performed using a Sysmex XN-1000v (Sysmex, Kobe, Japan) and a Beckman AU480 (Beckman, Brea, CA), respectively. At the study end point, EDTA blood was cryopreserved to create a stock of *P. fragile* iRBCs as previously described.^10^ Comprehensive tissue collection was performed after euthanasia. Aliquots of this stock are deposited with the Biodefense and Emerging Infections Research Resources Repository (BEI Resources; catalog no. MRA-1322).

### Histopathology.

Zinc formalin-fixed, paraffin-embedded and hematoxylin and eosin-stained terminal tissues were scanned on a Hamamatsu Photonics K.K. (Bridgewater, NJ) NanoZoomer360 slide scanner and analyzed by a veterinary pathologist. Quantification of hemozoin-containing blood vessels was conducted by manually identifying 100 blood vessels per tissue, quantifying those containing intact iRBCs characterized by hemozoin pigment, and reporting them as the number of iRBC-containing blood vessels per 100 blood vessels.

### *Plasmodium fragile* in situ hybridization.

In situ hybridization (ISH) was performed using an RNAscope (Advanced Cell Diagnostics, Newark, CA) kit with probes targeting *P. fragile *18S ribosomal RNA (rRNA; GenBank M61722). Four-micrometer tissue sections were mounted on Superfrost Plus Microscope slides (Fisher Scientific, Waltham, MA), baked for 3 hours at 60°C, deparaffinized in xylene and 100% ethanol, and then, dried at room temperature for 5 minutes. A Ventana Discovery Ultra automatic stainer (Roche, Basel, Switzerland) was used to conduct messenger RNA (mRNA) target retrieval (ACD, Newark, CA), endogenous peroxidase quenching (Roche), mRNA protease incubation (ACD), probe hybridization (ACD), signal amplifier hybridization (ACD), fluorescein isothiocyanate (Roche) color development, and 4’,6-diamidino-2-phenylindole (Fisher Scientific) counterstaining. Slides were then washed 10 times with alternating rounds of deionized (DI) water containing 0.1% Dawn dish soap (Procter & Gamble, Cincinnati, OH) and plain DI water followed by permanent mounting, overnight drying, digital imaging at 20× (Zeiss Axio Scan.Z1 slide scanner, Carl Zeiss Microscopy, Oberkochen, Germany), and analysis by a veterinary pathologist.

## RESULTS

### Clinical, hematological, and immunological parameters during experimental *P. fragile *infection.

Parasitemia was first measured by qPCR at 7 days postinfection (d.p.i.) and by Giemsa-stained thin blood smear at 15 d.p.i. (Table 1). Parasite staging with Giemsa staining revealed rings and trophozoites at 15 d.p.i. (5.9% and 6.6% of total iRBCs, respectively) and 17 d.p.i. (1.1% and 5.2% of iRBCs, respectively). Gametocytes and schizonts (0.5% and 0.3% of total iRBCs) were observed at 17 d.p.i. Hematological alterations indicative of clinical malaria, including anemia; increased white blood cell (WBC) count, red cell distribution width, and neutrophil, lymphocyte, eosinophil, and monocyte absolute counts; and decreased hemoglobin, red blood cell, and platelet counts, occurred by 7 d.p.i. and persisted through 17 d.p.i. (Table 1).^11^ Mean corpuscular volume, mean corpuscular hemoglobin, mean corpuscular hemoglobin concentration, mean platelet volume, and basophil counts were unchanged over time (Table 1). Clinical chemistry revealed increased serum aspartate aminotransferase and bilirubin indicative of hemolysis; however, taken together with the increased triacyl glyceride, these elevations may indicate hepatic dysfunction (Table 1).^12^^–^^14^ The clinical chemistry also revealed increased blood urea nitrogen and decreased serum albumin, which were interpreted together to indicate gastrointestinal (GI) bleeding (Table 1).^15^

**Table 1:** 

Clinical and Parasitic Parameters During *P. Fragile* Infection	0 d.p.i.	7 d.p.i.	15 d.p.i.	17 d.p.i	Reference Range*
Parasitemia					
Parasitemia, % iRBCs/total RBCs	0.0	0.0	15.8	8.6	0.0
*Plasmdium fragile *18s rRNA DNA copies/*µ*L DNA	0.0	3,310.63	761,432.2	141,496.0	0.0
Whole blood cell composition					
WBC count (×10³ cells/*µ*L)	4.92	5.73	6.9	8.08	3.65–14.34
RBC count (×10^6^ cells/*µ*L)^†^	6.33	5.72	5	4.03	5.09–6.52
Hemoglobin (g/dL)^†^	14.7	13.2	12.1	9.3	11.3–14.3
Hematocrit (%)^†^	42.9	39.4	34	27.1	36.0–45.0
Mean corpuscular volume (fL)	67.8	68.9	68	67.2	63.6–75.6
Mean corpuscular hemoglobin (pg)	23.2	23.1	24.2	23.1	20.3–24.0
MCHC (g/dL)	34.3	33.5	35.6	34.3	29.8–33.7
Platelet count (cells/*µ*L)^†^	437,000	354,000	245,000	196,000	240,000–565,000
Red cell distribution width (%)	13	13.2	15.8	15	11.4–15.6
Mean platelet volume (fL)	10.2	9.2	10.2	10.2	9.3–13.0
Leukocyte composition					
Neutrophils (×10^3^/*µ*L)	3.26	3.48	4.19	5.66	0.98–8.73
Lymphocytes (×10^3^/*µ*L)	1.32	1.71	1.96	2.26	0.94–5.69
Monocytes (×10^3^/*µ*L)^†^	0.27	0.44	0.7	0.16	0.04–0.58
Eosinophils (×10^3^/*µ*L)	0.05	0.05	0.02	0.00	0.00–0.54
Basophils (×10^3^/*µ*L)	0.02	0.05	0.03	0.00	0.00–0.05
Markers of liver function					
Alkaline phosphatase (U/L)	65	75	111	161	55–649
Alanine aminotransferase (U/L)	31	36	24	38	8–44
Aspartate aminotransferase (U/L)^†^	31	44	67	92	15–47
Albumin (g/dL)^†^	4.2	4	3	2.7	3.0–4.8
Globulin (g/dL)	2.7	2.8	2.8	2.9	1.9–3.9
Albumin/globulin ratio^†^	1.56	1.43	1.07	0.93	1.1–2.1
Total protein (g/dL)	6.9	6.8	5.8	5.6	6.1–7.6
Gamma-glutamyl transferase (U/L)	42	38	30	32	32–89
Markers of kidney function					
Total bilirubin (mg/dL)	0.12	0.21	0.49	0.62	0.10–0.70
Blood urea nitrogen (mg/dL)^†^	16.8	19.5	28.9	39.1	12.3–24.8
Creatinine (mg/dL)	0.91	0.87	0.97	0.92	0.32–1.05
Blood urea nitrogen/creatinine ratio	18	22	30	43	11–46
Markers of tissue damage					
Lactic dehydrogenase (U/L)^†^	761	651	991	1638	129–644
Cholesterol (mg/dL)	170	141	177	174	106–241
Triacyl glycerides (mg/dL)^†^	69	76	1,197	695	29–96
Serum chemistries					
Sodium (mEq/L)	151	148	144	145	144–154
Potassium (mEq/L)	4.4	3.9	3.8	3.2	3.4–4.3
Chloride (mEq/L)	108	109	104	104	104–112
Glucose (mEq/L)	50	62	56	68	44–95
Calcium (mg/dL)	8.9	8.7	8.4	6.5	9.4–12.2
Phosphorus (mg/dL)	5.9	4.1	2	6.3	3.4–7.5
Iron (*µ*G/dL)	118	100	105	59	50.0–212.0

d.p.i. = days postinfection; iRBC = infected red blood cell; MCHC = mean corpuscular hemoglobin concentration; RBC = red blood cell; rRNA = ribosomal RNA; WBC = white blood cell.

* Rhesus macaque reference ranges were derived from data from healthy rhesus macaques in the Tulane National Primate Research Center breeding colony. Values represent the upper and lower normal limits for each parameter.

^†^ CBC and blood chemistry out of reference range and altered in cases of human *falciparum* malaria.^11^^–^^15^

### Gross and histopathological effects of experimental *P. fragile* infection.

**Figure 1.**
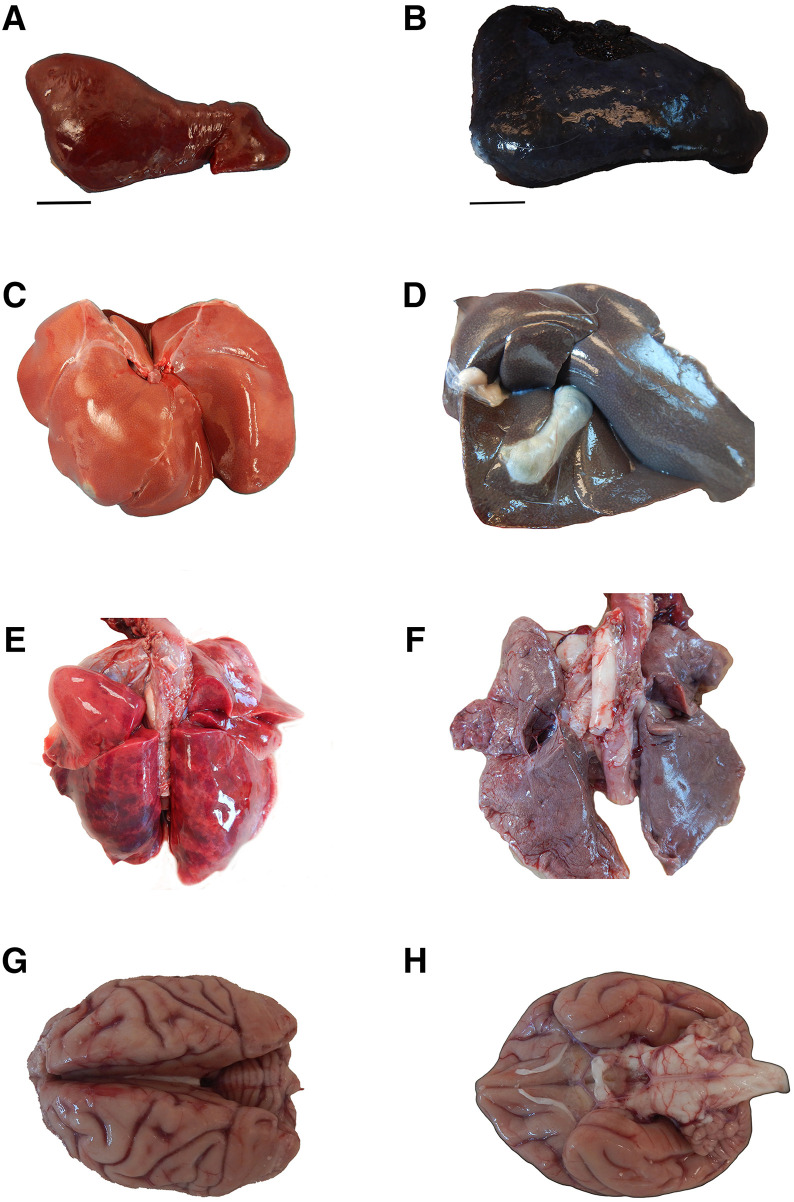
Gross pathological alterations of *Plasmodium fragile *infection of major organs. (**A** and **B**) Gross images of the spleen from an uninfected rhesus macaque (RM) and a *P. fragile-*infected RM, respectively. Bar = 1 cm. (**C** and **D**) Gross images of the liver from an uninfected RM and a *P. fragile-*infected RM, respectively. (**E** and **F**) Gross images of the lungs from an uninfected RM and a *P. fragile*-infected RM, respectively. (**G** and **H**) Gross images of the brain of a *P. fragile*-infected RM.

**Figure 2.**
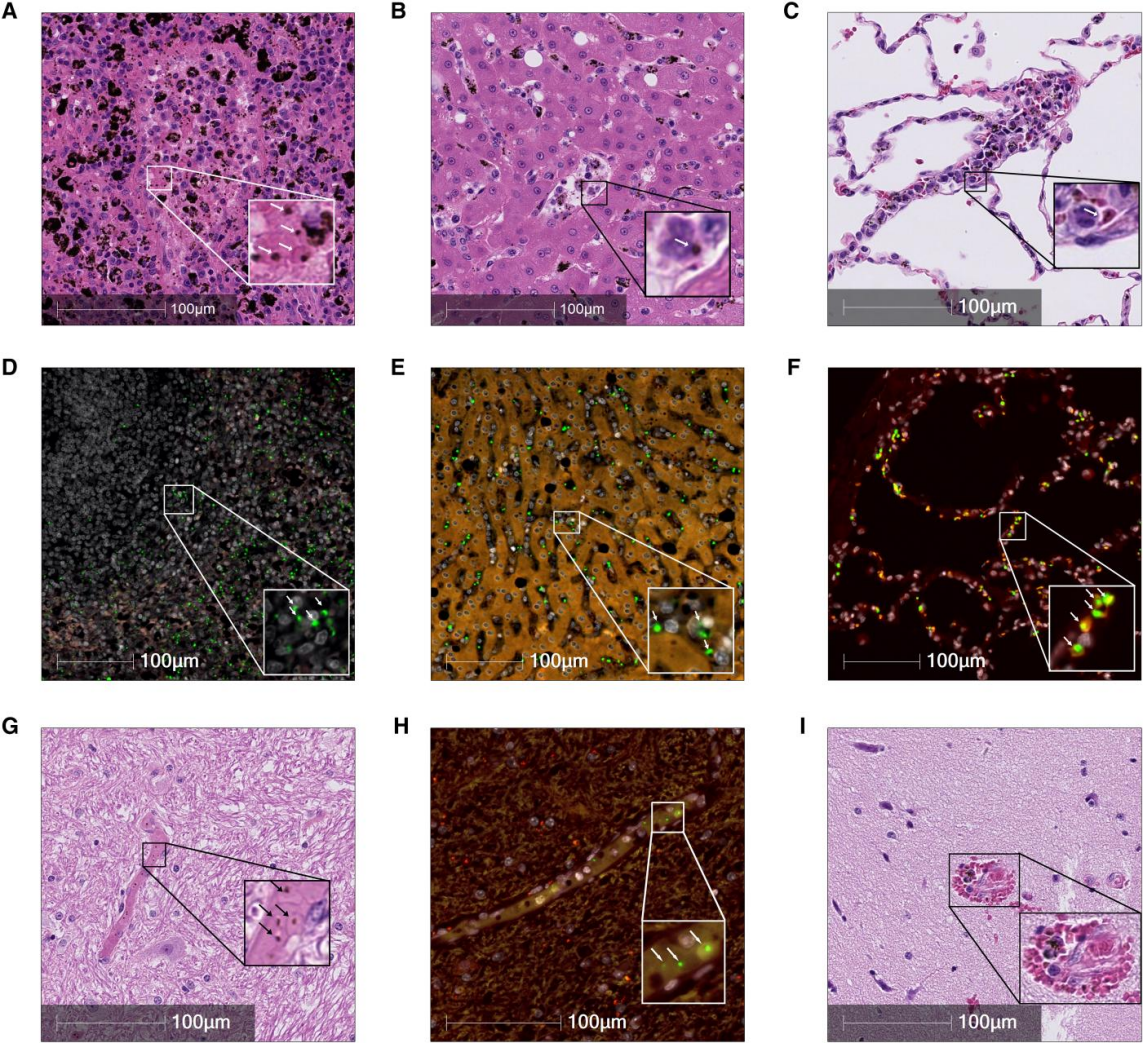
Representative histopathological alterations of *Plasmodium fragile *infection in major organs. (**A** and **D**) Hematoxylin and eosin-stained spleen tissue and *P. fragile* in situ hybridization (ISH) of spleen tissue after 17 days of *P. fragile* infection show the presence of *P. fragile *hemozoin or 18S ribosomal RNA (rRNA; arrows), respectively. (**B** and **E**) Hematoxylin and eosin-stained liver tissue and *P. fragile* ISH of liver tissue after 17 days of *P. fragile* infection show the presence of *P. fragile *hemozoin or 18S rRNA (arrows), respectively. (**C** and **F**) Hematoxylin and eosin-stained lung tissue and *P. fragile *ISH of lung tissue after 17 days of *P. fragile *infection show the presence of *P. fragile *hemozoin or 18S rRNA (arrows), respectively. (**G**) Brain capillary containing infected red blood cells (iRBCs) with hemozoin (arrows; cerebellum, hematoxylin and eosin). (**H**)* Plasmodium fragile *18S rRNA identified by RNAscope (arrows) in small brain capillary (cerebellum). (**I**) Microbleed coinciding with hemozoin-laden white blood cells and iRBCs (temporal lobe, hematoxylin and eosin). White indicates 4’,6-diamidino-2-phenylindole/nuclei. Green indicates *P. fragile *18S rRNA. Red/yellow indicates autofluorescence. All images have magnification of 40×.

Euthanasia was performed at 17 d.p.i when elevated parasitemia coincided with nonregenerative anemia. Gross postmortem findings were consistent with malaria-induced anemia, including splenomegaly with a dark red to purple coloration (Figure 1B). The liver was similarly darkened and friable (Figure 1D). The lungs were mottled pink to gray to purple (Figure 1F). The brain was grossly unremarkable (Figure 1G and H). Histopathologic findings included large amounts of hemozoin pigment and iRBCs within all tissues examined, including the liver, spleen, and lungs (Figure 2A–C). The presence of *P. fragile* in each tissue was further confirmed with RNAscope for *P. fragile* 18S rRNA (Figure 2D–F).

### Histopathological presentations of experimental *P. fragile* infection within distinct regions of the brain.

An aspect missing from prior studies using *P. fragile *as a model of *P. falciparum *is the effect of infection on the brain.^6^ *Plasmodium fragile *iRBCs were noted in all regions of the brain assayed. The cerebellum had the greatest burden (16% of all blood vessels containing iRBCs) followed by the parietal lobe (11%), the frontal lobe and medulla oblongata (10% each), the temporal lobe and basal ganglia (9% each), the occipital lobe (8%), and the pons (6%). Within the cerebellum, iRBCs were noted in small capillaries evidenced by hemozoin pigment and *P. fragile *18S rRNA observed by RNAscope (Figure 2G and H). Microhemorrhages containing iRBCs and pigment-laden leukocytes were observed in both the temporal cortex and the pons (Figure 2I).

## DISCUSSION

Here, we observed clinical hallmarks of malaria and detected the presence of all *P. fragile* intraerythrocytic life stages during experimental infection of a male RM. Furthermore, we observed increased WBC counts, including monocytes, after *P. fragile *infection. Alterations in relevant serum biomarkers indicated that *P. fragile *infection induced tissue damage similar to observations during severe *P. falciparum *malaria in humans.^12^^–^^14^^,^^16^ In human *P. falciparum *malaria, infection is associated with increased total leukocyte counts displaying elevated monocyte frequencies.^11^ Moreover, kidney damage, hepatic dysfunction, and GI damage are frequently observed in severe malaria and are associated with mortality.^15^^,^^17^ In this RM, hematological derangements indicated anemia, hepatic dysfunction, and GI bleeding; however, it is also important to note that euthanasia occurred before clinical disease progressed to advanced organ system failure as would be expected if left untreated.^15^^,^^17^

Histopathological examination of the lungs, liver, and spleen demonstrated the presence of iRBCs and hemozoin-containing leukocytes. The presence of *P. fragile* iRBCs in each tissue was confirmed through the detection of *P. fragile* ISH. In the brain, all sampled regions had capillaries containing iRBCs, consistent with prior observations in human cerebral malaria and in *P. coatneyi-*infected RMs.^18^^,^^19^ However, our histopathological assessment does not demonstrate the sequestration of *P. fragile *iRBCs that others have noted.^6^ Importantly, our observations of microhemorrhages within the temporal lobe and pons demonstrate that *P. fragile *infection is capable of causing cerebrovascular damage, similar to *P. falciparum *infection in humans.^20^

## CONCLUSION

In summary, *P. fragile* infection in a male RM mirrored *P. falciparum *malaria in humans, with similar alterations in serological, cellular, and immunological parameters. Our findings highlight the utility of the *P. fragile *NHP model for investigations focused on examining the mechanisms underlying severe malaria, particularly in vulnerable populations, such as pregnant individuals and children, that cannot be accomplished in humans because of practical and ethical constraints. Future work using this model should focus on characterizing the impact of *P. fragile* infection in larger groups with a range of ages and both sexes. Importantly, given our observation of *P. fragile* gametocytogenesis, examinations of severe *P. fragile* malaria in the context of vectored transmission are also feasible and warranted.
